# Trypanosomosis in The Gambia: prevalence in working horses and donkeys detected by whole genome amplification and PCR, and evidence for interactions between trypanosome species

**DOI:** 10.1186/1746-6148-4-7

**Published:** 2008-02-20

**Authors:** Gina L Pinchbeck, Liam J Morrison, Andy Tait, Joanna Langford, Lucinda Meehan, Saloum Jallow, Jibril Jallow, Amadou Jallow, Robert M Christley

**Affiliations:** 1 Faculty of Veterinary Science, University of Liverpool, Leahurst, Neston, CH64 7TE, UK; 2 Wellcome Centre for Molecular Parasitology, Glasgow Biomedical Research Centre, University of Glasgow, G12 8TA, UK; 3 Gambia Horse and Donkey Trust (GHDT), Sambel Kunda, Central River District, The Gambia

## Abstract

**Background:**

The Gambia has an increasing population of *equidae *largely used for agriculture and transportation. A review of cases at The Gambian Horse and Donkey Trust (GHDT) indicated that a common reason for presentation is a poorly defined medical condition often attributed to trypanosomosis. There are few reports describing the prevalence or the range of clinical signs associated with infection with different species of trypanosomes in horses and donkeys, but given the importance of these animals, the role of trypanosomosis requires investigation.

**Results:**

In total 241 animals from the Central River Division in The Gambia (183 horses and 58 donkeys) were screened using Whole Genome Amplification (WGA) followed by trypanosome species identification using polymerase chain reaction (PCR). The results indicated overall trypanosome prevalence of 91%; with an infection rate of 31% for *Trypanosoma congolense *Savannah, 87% for *Trypanosoma vivax *and 18% for *Trypanosoma brucei *sp. Multiple species were present in 43% of infections. Microscopy had a good specificity (100%) and positive predictive value (100%) for trypanosome detection, but the sensitivity (20%) and negative predictive value (10.5%) were low relative to PCR-based diagnosis.

Infection with *T congolense *showed the greatest negative effect on packed cell volume (PCV), while infection with *T. brucei *sp also had a significant, although lesser, negative effect on PCV. In addition, cases positive by microscopy were associated with significantly lower PCV. However, concurrent infection with *T. vivax *appeared to cause less effect on PCV, compared to animals infected with *T. congolense *alone.

**Conclusion:**

The prevalence of Trypanosomosis was high in both horses and donkeys. Infection with *T. congolense *appeared to have the greatest clinical significance, while *T. vivax *infection may be of limited clinical significance in this population. Indeed, there is evidence of *T. vivax *co-infection ameliorating the pathology caused by *T. congolense*. WGA and PCR allowed a more comprehensive analysis of field infections with the detection of infections below the threshold of microscopy, and provided indications of interactions between parasite species that would otherwise remain undetected. The study raises important questions about the epidemiology of trypanosome infection in relation to disease that require a full scale longitudinal analysis.

## Background

The Gambia is a small, sub-Saharan country located on the West Coast of Africa. A short, tropical rainy season begins in July and ends in September and a long dry season extends from October to June. Animal traction plays a significant role in the intensification of crop production and, in addition, *equidae *are used for transportation of goods, harvests and people over long distances. The 1974 National Agricultural Sample Survey estimated a population of 10,500 donkeys and 5000 horses. There has been a > 400% increase in the number of *equidae *over the 28 year period to 2002, when the census recorded 22,236 horses and 42,268 donkeys (Dr Kebba Daffeh, *pers comm*).

The pathogenic species of trypanosome found in The Gambia include *Trypanosoma congolense*, *Trypanosoma vivax *and *Trypanosoma brucei*, which are transmitted cyclically by tsetse flies (*Glossina *spp), although *T. vivax *can also be transmitted mechanically by biting flies [1]. The prevalence of trypanosomosis reported in Gambian *equidae *has primarily been determined using classical parasitological methods [2-4]. In general, horses are considered to be highly susceptible to infection, while donkeys are considered to be more resistant, although the reasons for lower prevalence in donkeys could also be due to feeding preferences of the tsetse vector. Snow *et al*. [3] reported that given the susceptibility of *equidae *and reported foaling rates, the numbers of animals in The Gambia must be maintained by import from areas of lower challenge, while other authors suggest that the level of tsetse challenge has reduced as a result of changes in the habitat. The data on prevalence do not seem to support these views as the surveys undertaken in 1989–91 show a prevalence of 6–10% in horses and 9.2% in donkeys [2,3], which does not seem to be consistent with a very high challenge, whereas studies undertaken in the mid to late 1990s show an increased prevalence with values ranging from 43–63% in horses and 6.2–43% in donkeys [4,5], suggesting that the challenge has increased. As all these studies used the buffy coat method of diagnosis [6] the differences cannot be methodological. However, one of the most recent studies [4] was undertaken on animals attending a veterinary clinic, which could, in part, explain the higher prevalence. In each of the studies reported to date, the species of infecting trypanosome were defined; with a general conclusion that *T. congolense *is the most prevalent followed by *T. vivax *with *T. brucei *being rare, although the prevalence of *T. vivax *was shown to be higher in the dry season. There has been a significant amount of work carried out developing more sensitive PCR based methods for detecting the trypanosome species infecting livestock. Whereas the buffy coat technique has a threshold of detection of around 1 × 10^2 ^parasites/ml [6], PCRs have been developed that target multi-copy targets, such as the satellite repeats on the minichromosomes [7] or the Internal Transcribed Spacer (ITS) of ribosomal DNA [8], the sensitivity of which are simply limited by the presence of trypanosome DNA in the sample i.e. a single trypanosome. For a comprehensive review of these techniques, see [9]. Furthermore, in a recent study from The Gambia, it was shown that the number of detected infections was seven times higher using PCR [5].

The Gambian Horse and Donkey Trust (GHDT)[10] is a small non-governmental organisation (NGO) based in Sambel Kunda in the Central River District of The Gambia. A previous review of clinical cases presented to clinics (Table 1) indicated that one of the most common reasons for presentation is for a poorly defined medical condition with signs including weakness, dullness, weight loss, anorexia and occasional presence of dependant oedema; these signs are often attributed by the owners to trypanosomosis.

**Table 1:** Top ten presenting complaints recorded in 358 horses and donkeys presenting to The Gambia Horse and Donkey Trust April – Sept 2005 (data extracted from GHDT clinical database).

Presenting complaint	Number of cases(n = 358)	% of cases
Loss of appetite	127	35
Weak	121	34
Suspect Trypanosome infection	97	27
Overwork/immaturity	56	16
Poor condition	53	15
Feet	47	13
Respiratory	44	12
Eyes	30	8
Diarrhoea	16	4
Teeth	15	4

The aims of this study were to estimate the prevalence of trypanosomes in both clinically presented and asymptomatic horses and donkeys in both the wet and dry season, as well as determining which species of trypanosomes might be responsible. The development of highly sensitive, species specific PCR based methods of diagnosis [9] provides the possibility of more comprehensive data on prevalence. This allowed the testing of previous conclusions based on less sensitive methodology, as well as the investigation of interactions between species in the development of clinical disease. The results provided a series of important findings that challenge some of the conclusions of previous studies, and provide justification for more extensive future investigation into the epidemiology in order to inform control and treatment strategies.

## Results

A total of 241 animals (183 horses and 58 donkeys) were sampled. Forty four animals were diagnosed positive for trypanosome infection by microscopy, a prevalence of 18.25%. Of the 44 animals microscopically identified as positive, speciation was undertaken using trypanosome-specific PCR after Whole Genome Amplification (WGA). This technique is based on the replication of large stretches of double stranded DNA by strand displacement and random hexamer primers resulting in the amplification of the target DNA. These results indicated that 29 animals were infected with *T. congolense *Savannah, 39 with *T. vivax *and 17 with *T. brucei *sp (33/44 animals had mixed infections). All samples were negative for *T. congolense *Forest (therefore hereafter all references to "*T. congolense*" allude to *T. congolense *Savannah). When WGA and PCR analysis was carried out on the microscopically negative samples, the PCR results indicated that 219/241 were positive indicating a prevalence of trypanosome infection in the population as a whole of 91% (Table 2). Mixed infections with more than one trypanosome species were common with 95/219 (43%) animals infected by more than one species. Therefore, our results indicate that, compared to PCR, microscopic examination had good specificity (100%) and positive predictive value (100%) for trypanosomes in this population. However the sensitivity (20%) and negative predictive value (10.5%) were low, indicating a high frequency of false negatives results. The increased power of detection by PCR is very apparent, and suggests a high level of infection at low parasitaemia that was undetectable by microscopy.

**Table 2:** Prevalence of trypanosome species in 241 horses and donkeys from the Central River Division, The Gambia 2006.

	Dry season (n = 154)		Wet Season (n = 87)		Horses (n = 183)		Donkeys (n = 58)		Overall prevalence
	N	Prev %^‡^	N	Prev %	N	Prev %	N	Prev %	% (95%CI)

*T. congolense*	44	29	30	34	53	29	21	36	31 (25, 37)
*T. vivax*	131	85	79	91	165	90^†^	45	78^†^	87 (83, 91)
*T. brucei *sp	25	16	19	22	29	16^†^	16	28^†^	18 (13, 23)
*T. vivax *+ *T. congolense *only	32	20	19	22	36	20	15	26	21 (16,26)
*T. congolense *+ *T. brucei *sp only	1	1	0	0	0		1		0.5(0,1)
*T. vivax *+*T. brucei *sp only	18	12	10	11	18	10	10	17	12 (8, 16)
*T. vivax *+ *T. congolense *+*T. brucei *sp	5	3	10	11	11	6	4	7	6 (3,9)
Overall positive for any species of trypanosome	139	90	80	92	171	93^†^	48	83^†^	91 (87, 94)

*T. congolense Savannah, *T. vivax*, and *T. brucei *sp figures are the overall % positive (including mixed infections). Mixed infections are for the stated trypanosome species only.

^† ^Significantly different at P = 0.05

^‡ ^Prev = prevalence (rounded to whole numbers)

Although the prevalence of all *Trypanosoma *species was slightly higher in the wet season (92%) compared to the dry season (90%) this difference was not significant (P = 0.7) for any of the species or for mixed infections (Table 2). The prevalence in the 73 samples from asymptomatic animals sampled in surrounding villages (87%) was not significantly different to that in animals presented to GHDT clinics (92%). There was a higher overall prevalence in horses (93%) compared to donkeys (83%) (Table 2) and horses had a significantly higher prevalence of *T. vivax *infection. However, significantly more donkeys had *T. brucei *sp infections and donkeys had a greater proportion of mixed infections compared to horses.

The presenting clinical signs of infected animals were variable ranging from lethargy and anorexia reported by the owner, with no abnormalities detected on clinical examination, to marked pyrexia, tachycardia and tachypnoea. Dependant oedema was an occasional finding in 27 positive and 3 negative animals (diagnosed by PCR). The majority of animals had a body condition score (BCS) of 2 or lower. However there was no significant difference in BCS, total protein or other recorded clinical parameters between animals with and without trypanosome infection diagnosed by PCR. However, 91% of animals were positive for trypanosome infection and a study with larger numbers of uninfected animals is required to evaluate clinical signs associated with infection.

Animals with *T. congolense *or *T. brucei *sp had a significantly lower PCV than non-infected animals (P < 0.01), but *T. vivax *infection did not appear to affect the mean PCV. The results of the multivariable linear regression model, to investigate the effect of different *Trypanosoma *species on PCV, are shown in Table 3. Infection with *T. congolense *had the greatest negative effect on PCV. However concurrent infection with *T. congolense *and *T. vivax *appeared to cause less effect on PCV when compared to infection with *T. congolense *alone in terms of the effect on PCV, as evidenced by a significant interaction term with a large positive coefficient (Table 3). Co-infection with *T. brucei *sp also had a small negative effect on PCV. A positive diagnosis by microscopic examination was associated with a significantly lower PCV, possibly reflecting the effect of the level of parasitaemia. In addition, donkeys showed a lower PCV relative to horses, and female animals had a lower PCV relative to males.

**Table 3:** Multivariable linear regression model of the effect of infection with different *Trypanosoma *species and other significant confounding factors on the packed cell volume (PCV) of 241 horses and donkeys from the Central River Division, The Gambia 2006.

Variable		Coefficient	± S.E	*P-value*
Intercept		36.1	1.2	
*T. congolense*				
	-ve	Ref		
	+ve	-11.9	0.8	*<0.001*
*T. Brucei *sp				
	-ve			
	+ve	-2.0	0.9	*0.03*
*T. vivax*				
	-ve	Ref		
	+ve	-3.3	1.2	*0.007*
Gender				
	Male	Ref		
	Female	-1.6	0.7	*0.03*
Species				
	Horse	Ref		
	Donkey	-1.9	0.8	*0.02*
*Trypanosoma *status on microscopic examination				
	-ve	Ref		
	+ve	-5.3	1.0	*<0.001*
T. congolense* T. vivax interaction term				
		Ref		
	*+ve*	*6.4*	*2.3*	*0.006*

Ref – Reference category

Model diagnostics showed that the assumptions of normality were met and removal of observations with a large influence showed the model to be stable.

## Discussion

This study found the prevalence of trypanosomosis in horses and donkeys in the Central River Division of The Gambia by microscopy was 18.25%, a rate of detection similar to that seen in other studies by microscopy in this region (7.5 – 34.2%[5]). Although speciation was not carried out under microscopy, accurate speciation was undertaken using species specific primers [7]. This allowed the detection of mixed infections much more accurately, as it is not dependent on a patent parasitaemia. The prevalence as determined by species specific PCR after Whole Genome Amplification (WGA) on the samples was 93% and 83% in horses and donkeys, respectively. These values are considerably higher than any of the previously published figures, which range from 7% [3] to 61% [4], depending on the region of the country sampled, the population sampled and the time of year. However, the published data are largely based on microscopy, which from this study and that of Faye *et al*. [5] exhibits a much lower sensitivity. The sensitivity of PCR based methods has been reviewed [9] and these methods can detect as few as 1–20 trypanosomes/ml, although the exact sensitivity depends in part on whether prior concentration of trypanosomes is undertaken before PCR amplification. We have previously estimated the sensitivity of whole genome amplification followed by PCR, the technique used in this study and, using the satellite PCR, can detect 100 trypanosomes/ml from blood spotted on FTA filters [11]. Similar to the work by Dhollander *et al*. [4], the majority of animals sampled in this study were those presented for clinical problems to the mobile or home clinic, and therefore, the prevalence estimates are unlikely to be representative of the general population. However, the prevalence in this study for the samples from apparently healthy animals sampled in surrounding villages was also very high (87%). The use of target repetitive sequences in PCR to detect *T. congolense *and *T. vivax *has to be treated with an element of caution, since it has been suggested that PCR using these highly repetitive elements can amplify from DNA that remains in circulation for some time after parasite death [5], and does not necessarily indicate active infection. This explanation is unlikely to be the case for the single copy microsatellite used for *T. brucei *sp detection, as the sensitivity of the PCR is much lower [11]. The minimum conclusion from a positive result using the satellite repeat PCRs is that the animal either is, or has been infected with *T. congolense *and *T. vivax *at some point. In every case the possibility of cross-contamination was kept to a minimum, with positive and negative controls included to ensure no contamination, and by processing each sample individually. The main difference between the present study and that of Faye *et al*. [5], where PCR was also utilised on samples from the same geographic region, is the higher detection of *T. vivax *(87%) and *T. brucei *sp (18%) cases. Faye and colleagues demonstrated a much lower prevalence of *T. vivax *(21%) compared to our results, despite using the same oligonucleotide primers, although it must be pointed out that their sample number was comparatively low (11 horses and 29 donkeys). In our study the use of WGA, which increases overall sensitivity by replicating the genomes present in a particular sample [11], may explain the increase in sensitivity by increasing the detection level of trypanosomes at low parasitaemia above the threshold of PCR, thereby explaining the discrepancy in prevalence between the two studies. In conclusion, the prevalence of both *T. vivax *and *T. brucei *sp reported in this study are much higher than previously reported, and the use of WGA followed by PCR is likely to be the explanation for this.

From our study, *T. congolense *Savannah is associated with a lowered PCV (Table 3), similar to previous studies. For example, Dhollander *et al*. [4] reported a prevalence rate of 63%, of which 64% were infected with *T. congolense *and 32% *T. vivax*. The majority of these animals were also anaemic, and the correlation between an animal being microscopically positive and anaemic has also been demonstrated [2]. Therefore the results of Dhollander and others correlate with our results, in that the microscopically positive cases had more severe anaemia. PCR allows the detection of subpatent parasitaemias, such as those characteristically resulting from *T. vivax *and *T. brucei *sp, and therefore results in a much more comprehensive and realistic picture of what is present. However, microscopic positivity is also associated with the acute stage of disease where parasite burden is greater, and it may be that this stage is more acute in *T. congolense*, resulting in a greater degree of presentation at clinics. Our results raise an important issue however, as they suggest that the association of particular parasite species with particular pathology may only be accurately determined in field studies if the true parasite prevalence's are known. The assumption that *T. congolense *causes the more severe disease may be correct, but as is indicated by our results there are added complexities, such as the pathogenic influence of *T. brucei *sp, and perhaps most intriguingly, the ameliorative effect of concurrent *T. vivax *infection. However, a more comprehensive and controlled study is required to fully understand the epidemiology of the disease, and the roles of different trypanosome species, mixed infections, and different host species in the holistic picture of trypanosomosis.

In the case of *T. brucei *sp, it has been reported that infection in *equidae *is severe and often fatal [12], and this has been suggested as a reason for the few *T. brucei *sp cases observed, due to the rapid death of the host [4]. If this scenario is correct, then our prevalence of *T. brucei *sp infection of 18% is likely to be an underestimate and the clinical effect may be more severe. Alternatively *T. brucei *sp is not as virulent in *equidae *as previously thought, or there are variations in pathogenesis induced by different strains of *T. brucei *sp. In the *equidae *examined in our study, infection with *T. brucei *sp was associated with a significantly lower PCV, although this effect was not as great as with *T. congolense *infections. In contrast, although the prevalence of *T. vivax *was very high, it did not appear to be associated with a decreased PCV, which seems to confirm the notion of *T. vivax *being relatively apathogenic in equines [12-14]. However, experimental infection in horses with subsequent disease has been demonstrated [15], and in cattle *T. vivax *infections have been reported as both severe, causing pyrexia, and of a more chronic nature with progressive anaemia and weakness [14]. Certainly strain-dependent pathology does occur in *T. vivax*, with a haemorrhagic strain reported in cattle [16]. Interestingly, the multivariable model indicated that concurrent infection with *T. congolense *and *T. vivax *is associated with a reduced expression of pathology (in terms of PCV) relative to infections with *T. congolense *alone, and this may be due to competition between the trypanosome species. The phenomenon of competition was initially identified in superinfections of *T. congolense *[17,18], where primary infections prevented establishment of a second strain. It was also investigated between species [19], where goats already infected with *T. congolense *delayed the establishment of a *T. brucei *secondary challenge, but not that of *T. vivax*. Therefore, one could postulate from our data that prior or co-infection with *T. vivax *ameliorates the pathology due to *T. congolense *by a similar interference phenomenon. Obviously, more detailed analysis of populations, and controlled surveys, ideally using naïve young animals, are desirable to analyse whether this phenomenon is occurring, and if it is, what implications this has for the epidemiology of the disease.

Previous studies have demonstrated that trypanosome prevalence is higher in horses then in donkeys [4,5], although Faye *et al*. [5] included only a small numbers of horses. To explain these findings it has been suggested that donkeys are inherently resistant to trypanosomes, that there is a feeding preference of tsetse flies for horses, and that donkeys have a better ability to deter flies from feeding by skin rippling, head movements and other behavioural avoidance mechanisms [2]. In the current study there was a slight overall difference in prevalence between horses and donkeys, although *T. vivax *was more prevalent in horses, and *T. brucei *and mixed infections were more prevalent in donkeys. These results, assuming that a single species of tsetse fly is involved in transmission, argue against feeding preference or behavioural differences. In the present study donkeys had a small but significantly lower PCV than horses. There are few published reference values for PCV in working horses and donkeys, although Pritchard *et al*. [20] found mean values for PCV were low, but not significantly different in both (uninfected) working horses and donkeys. This may be due to mal- and under-nutrition, as well as infectious diseases.

## Conclusion

In summary, trypanosome infection is highly prevalent in *equidae *in the Central River Division of The Gambia, and appears to be a clinically significant condition associated with anaemia. There were not any reliable, pathognomic clinical signs to definitively aid diagnosis. The sensitivity of microscopy for diagnosis was poor, and analysis by PCR following Whole Genome Amplification revealed the prevalence of trypanosome infection to be startlingly high and much higher than previously reported. The increase in detection seen in our study is very likely due to the combination of WGA and PCR. The infections were predominated by *T. vivax *(87%), followed by *T. congolense *Savannah (31%) and *T. brucei *sp (18%). There were also a high proportion of mixed infections. PCV was significantly lower in animals infected with *T. congolense *or *T. brucei *sp and, interestingly, there appeared to be clinical differences between those animals concurrently infected with *T. congolense *and *T. vivax *compared to those infected with *T. congolense *alone in terms of the effect on PCV. Therefore, the epidemiology of trypanosomosis in the field with interactions between different trypanosome species and strains in different species of host is obviously complex, but the use of tools such as WGA and PCR allow deeper analysis by allowing detection of subpatent infections, and therefore a more comprehensive picture of the dynamics of parasite populations in the host. However, in order to fully analyse the situation in the case of African trypanosomes, a comprehensive and controlled longitudinal study is necessary in order to fully examine the influence of different host species, different trypanosome species and environmental factors. Furthermore, data on these aspects are particularly required in order to qualitatively inform control and treatment strategies.

## Methods

### Sampling design

Two cross-sectional studies were conducted in the Central River District of The Gambia in March (dry season) and August (wet season) of 2006; each was conducted over a 4 week period. Horses and donkeys sampled were those presented either at the GHDT home clinic, or at mobile clinics held at local markets, for any medical problem. The presenting medical condition defined by the owner varied enormously, including; 'suspected trypanosomes'; lethargy; anorexia; weight loss. In addition, a random sample was performed in 15 surrounding villages (total 73 animals); up to 5 animals were selected from all those present in the village at the time of the visit.

The following clinical parameters were recorded for each animal; heart rate, respiratory rate, temperature, mucous membrane colour and capillary refill time, body condition score (on a scale of 0–5; 0 = very poor, 5 = very fat) [21], girth and length measurements for weight calculation, and the presence or absence of dependant oedema. Details of ownership, reason for presentation, home village, and age (by dentition) were also recorded.

### Blood collection

A jugular blood sample was collected into 5 ml ethylene diamine tetra-acetic acid (EDTA) coated vacutainer tubes, and from this the blood measurements of PCV, total protein (TP) and parasitological status were carried out. Parasitological examination included microscopic examination of whole blood and of blood films from the microhaematocrit buffy coat [6]. Where possible, samples were processed immediately after sampling in the field, using a 'Spindoctor™' manual centrifuge and a solar-powered, portable microscope (Diamedica Limited, UK). Speciation of trypanosomes by microscopy was not attempted. A 200 μl aliquot of all samples was also dried onto FTA filters (Whatman, UK) and stored prior to Polymerase Chain Reaction (PCR) analysis to detect and speciate trypanosomes.

### Molecular Characterisation

Five 2 mm discs from each FTA filter were washed (4× 200 μl FTA buffer, 3 × 200 μl TE buffer pH 8.0), and a Whole Genome Amplification (WGA) reaction carried out, using the Genomiphi^® ^kit (Amersham) as described previously [11]. This technique uses a bacterial polymerase to amplify large stretches of DNA by strand displacement using random hexamer primers [22]. Speciation PCRs for *T. congolense *Forest and Savannah, and *T. vivax *were carried out using species-specific oligonucleotide primers directed against the 170 bp satellite repeats [7]. However this PCR for *T. brucei *sp showed some cross reaction with *T. vivax*, so *T. brucei *speciation was undertaken with a nested microsatellite primer, PLC [23]. Verification of speciation was also carried out using different primers for *T. vivax *that target a species-specific antigen [24], and primers directed against the internal transcribed spacer (ITS) region of ribosomal DNA [25]. The satellite repeat PCRs only amplify a product from DNA of the respective trypanosome species, whereas the ITS primers are universal across trypanosome species, but give products of different sizes distinctive for each species.

### Data analysis

Prevalence estimates with 95% confidence intervals were calculated and Pearson Chi-square tests were used to compare the prevalence in different groups of animals. T-tests were used to compare continuous variables. In order to assess the effect of infection with different trypanosome species on the PCV, whilst allowing for other confounding variables (age, gender, species and whether the animal was presented at the clinic or was from village sampling), multivariable linear regression was used. Infection status for all 3 species of trypanosome and other variables with p < 0.25 in initial univariable screening were considered for inclusion in the multivariable linear regression model, built using backward elimination procedures. Significant variables (p < 0.05) or variables that improved the fit of the model based on the Akaike information criteria statistic were retained in the model. Two-way interaction terms were used to test the association between infection with more than one species of Trypanosome and PCV. Significant interaction terms were retained in the model. The fit of the model was assessed by examining plots of the residuals versus the fitted values and of normal quantile plots of the residuals to test the assumption of normality of the residuals. Finally, Cook's distance plots were examined to assess the influence of individual observations on the regression coefficients. All analyses were performed using the software packages Minitab (Minitab 14, Minitab Inc., Pennsylvania, USA) and S-Plus (S-plus 2000, Mathsoft Inc, Cambridge, Mass, United States).

## Authors' contributions

GP and RC conceived and supervised the project. GP, RC and JL collected the data and samples in March 2006. LM collected those from August 2006. SJ, JJ and AJ organised collection of samples at both time points, and provided logistical support in The Gambia. LM and AT carried out the WGA and PCR analysis and GP the statistical analysis. All read and approved the final manuscript.
